# *Solanum elaeagnifolium* and *S*. *rostratum* as potential hosts of the tomato brown rugose fruit virus

**DOI:** 10.1371/journal.pone.0282441

**Published:** 2023-03-01

**Authors:** Maor Matzrafi, Jackline Abu-Nassar, Chen Klap, Meital Shtarkman, Elisheva Smith, Aviv Dombrovsky

**Affiliations:** 1 Department of Plant Pathology and Weed Research, Newe Ya’ar Research Center, Agricultural Research Organization (ARO)–Volcani Institute, Ramat Yishay, Israel; 2 The Robert H. Smith Faculty of Agriculture, Food and Environment, The Hebrew University of Jerusalem, Rehovot, Israel; 3 Department of Plant Pathology and Weed Research, Agricultural Research Organization (ARO)–Volcani Institute, Rishon LeZion, Israel; ICAR - Indian Institute of Horticultural Research (IIHR), INDIA

## Abstract

Invasive weeds cause significant crop yield and economic losses in agriculture. The highest indirect impact may be attributed to the role of invasive weeds as virus reservoirs within commercial growing areas. The new tobamovirus tomato brown rugose fruit virus (ToBRFV), first identified in the Middle East, overcame the *Tm-2*^*2*^ resistance allele of cultivated tomato varieties and caused severe damage to crops. In this study, we determined the role of invasive weed species as potential hosts of ToBRFV and a mild strain of pepino mosaic virus (PepMV-IL). Of newly tested weed species, only the invasive species *Solanum elaeagnifolium* and *S*. *rostratum*, sap inoculated with ToBRFV, were susceptible to ToBRFV infection. *S*. *rostratum* was also susceptible to PepMV-IL infection. No phenotype was observed on ToBRFV-infected *S*. *elaeagnifolium* grown in the wild or following ToBRFV sap inoculation. *S*. *rostratum* plants inoculated with ToBRFV contained a high ToBRFV titer compared to ToBRFV-infected *S*. *elaeagnifolium* plants. Mixed infection with ToBRFV and PepMV-IL of *S*. *rostratum* plants, as well as *S*. *nigrum* plants (a known host of ToBRFV and PepMV), displayed synergism between the two viruses, manifested by increasing PepMV-IL levels. Additionally, when inoculated with either ToBRFV or PepMV-IL, disease symptoms were apparent in *S*. *rostratum* plants and the symptoms were exacerbated upon mixed infections with both viruses. In a bioassay, ToBRFV-inoculated *S*. *elaeagnifolium*, *S*. *rostratum* and *S*. *nigrum* plants infected tomato plants harboring the *Tm-2*^*2*^ resistant allele with ToBRFV. The distribution and abundance of these *Solanaceae* species increase the risks of virus transmission between species.

## Introduction

Weeds are a source of significant agricultural crop yield and economic loss. Worldwide, the potential yield loss of major agriculture crops due to weeds (34%, on average) is higher than other crop pests, including insects, pathogens, viruses and animal pests [1]. Among weeds, invasive species present major economic and ecological threats to agriculture and natural areas. In recent decades, there has been a rise in reports of invasive weed species due to significant manmade global changes. Among the leading causes for this trend are import-export trade [2, 3] and climate change [4, 5]. In the United States alone, annual losses caused only by crop-related invasive weeds were estimated to be more than $27 billion dollars [6]. In Israel, several invasive species such as *Parthenium hysterophorus*, *Solanum* spp., *Ambrosia confertiflora* and *Amaranthus* spp. have been documented [7]. Apparently, the invasion route of these weeds is via imported animal feed shipments [7]. The damage due to invasive weed species is not limited to yield losses, but could be associated with the increased spread of fire-fuel [8–10] and strong allergenic effects [11–13]. Although it may be underestimated, the greatest indirect impact of invasive weeds on crop yield may be attributed to their role as virus reservoirs within commercial growing areas.

Several weeds have been previously identified as potential reservoirs of plant viruses such as iris yellow spot virus, potato leafroll virus and potato virus Y (PVY) [14]. Various studies have shown that within identified weed virus hosts there was a high proportion of invasive weeds. *P*. *hysterophorus*, a prominent invasive wide spread weed species, was infected with cherry tomato leaf curl virus and tobacco curly shoot alpha satellite [15]. In Turkey, *A*. *retroflexus* appeared to be a common host of several viruses including cucumber mosaic virus, PVY and tomato spotted wilt orthotospovirus [16]. *Ventenata dubia*, an invasive weed species infesting grasslands, rangelands and pastures throughout the USA was susceptible to barley or cereal yellow dwarf virus infection and served as a transient agent for crop infections [17]. In Israel, we documented new invasive weed species such as *A*. *graecizans* and *A*. *muricatus* as optional hosts of tobamovirus cucumber green mottle mosaic virus [18].

The new tobamovirus tomato brown rugose fruit virus (ToBRFV), first identified in the Middle East [19, 20], overcomes the *Tm-2*^*2*^ resistant allele of cultivated tomato varieties grown trellised in greenhouses [19]. Outbreaks of the ToBRFV disease were later reported in North America [21, 22], Germany [23], Turkey [24], Greece [25], Spain, the Netherlands and China [26]. ToBRFV causes a range of symptoms in tomato [27]. Fruit yellowing and bleaching are the most commonly occurring symptoms, accompanied by mottled mosaic leaves [19, 28]. Concomitantly, a worldwide spread of the mechanically transmitted potexvirus, pepino mosaic virus (PepMV), has also occurred [29–32]. PepMV causes fruit necrosis and plant wilting [33, 34]. ToBRFV and PepMV profoundly affect the yield and quality of tomato plants.

PepMV and tobamoviruses are seed-borne, mechanically transmitted viruses [29, 35]. Plant manipulations such as planting, fruit picking, pruning and trellising, essential for tomato plant cultivation, are practices predisposing plants for disease spread of mechanically transmitted viruses. Regarding PepMV, even direct contact between leaves of healthy and infected plants can spread the disease [30]. Beneficial insects are also implicated in transmission of ToBRFV, which could occur through mechanical adsorption of the virus to the insects [36, 37]. Partial host plant analyses for ToBRFV identified various weed species of genus Solanum, such as *S*. *nigrum*, serologically positive for the virus [19, 38]. Susceptibility of *S*. *nigrum* to PepMV infection is well documented [39, 40].

Recently a synergism has been documented between ToBRFV and the mild strain PepMV-IL, which were both found in mixed infections of commercially available tomato fruits and in elite tomato crops in Israel [41, 42]. The new severe disease symptoms, primarily observed during the wintertime in Israel, were characteristic of an aggressive PepMV strain, although only the mild PepMV-IL was present in the mixed infected plants [42]. The synergism between ToBRFV and PepMV-IL was manifested in an increased PepMV-IL titer in the presence of ToBRFV, when compared to PepMV-IL titer in singly infected tomato plants [42].

The main goal of the current study was to determine the potential of invasive weeds as hosts of ToBRFV and PepMV-IL. Invasive weeds common within commercial tomato growing areas (e.g., in greenhouses and open fields) were tested. Identifying ToBRFV and PepMV-IL potential weed hosts will contribute to more efficient disease management practice for weed control throughout the tomato production cycle.

## Materials and methods

### Plant materials and virus sources

Seeds of weed species collected during 2018–2019 from agricultural fields or field margins were tested for susceptibility to ToBRFV and PepMV-IL infection. Tested species were: *A*. *blitoides*, *A*. *retroflexus*, *Conyza bonariensis*, *C*. *canadensis*, *Digitaria sanguinalis*, *Setaria adhaerens*, *S*. *elaeagnifolium*, *S*. *nigrum*, *S*. *rostratum*, *Sorghum halepense*, *Xanthium strumarium*. Seeds were germinated in 500 ml pots filled with commercial potting media (Tuff, Marom Golan, Israel) including Osmocote® slow release fertilizer. Seedlings were grown in a greenhouse under natural growing conditions during the spring season at Newe Ya’ar Research Center. At a three to four leaf stage, seedlings were transferred into 300 ml plastic pots.

ToBRFV and PepMV-IL were extracted from infected tomato plants harboring the *Tm-2*^*2*^ resistant allele. To ensure single inoculations of either ToBRFV or PepMV-IL, ToBRFV was isolated on *Nicotiana tabacum* cv. Samsun, systemically infected by ToBRFV only (Oded Lachman, personal communication). PepMV-IL was isolated on *D*. *stramonium* plants, systemically infected by PepMV which developed necrotic local lesions towards ToBRFV [19, 43]‏. Cultures of each virus were propagated continuously on tomato plants cv. Ikram (heterozygotic for the *Tm-2*^*2*^ resistance allele), serving as an inoculum source. The inoculum was prepared by grinding virus-infected tomato leaves ~1 g/25 ml 0.01M sodium phosphate buffer pH 7.0. For estimation of viral content, viral proteins were extracted with urea-SDS-β-mercaptoethanol buffer using a dilution factor of 1.6, and proteins were resolved on SDS-PAGE adjacent to a bovine serum albumin control. Coomassie staining allowed estimation of viral CP in the inoculum, which was ~0.2 mg/ ml sap inoculum of ToBRFV and ~0.05 mg/ml sap inoculum of PepMV-IL. Inoculations were performed by rubbing the ToBRFV or PepMV-IL sap extract on the leaves of weeds, using 5–10 plants per species. Experimental inoculations of ToBRFV onto *S*. *elaeagnifolium* and *S*. *rostratum* plants were repeated using 34 and 26 plants, respectively. Plants were kept in a growth chamber at 24 ± 3°C. At ca. thirty days post inoculation (dpi) leaf samples were collected for virus diagnostics using ELISA and RT-PCR tests.

### Viral inoculations and bioassays

*S*. *elaeagnifolium*, *S*. *rostratum*, *S*. *nigrum* and *S*. *lycopersicum* plants bearing the *Tm-2*^*2*^ resistance allele were grown in chambers in a glasshouse under controlled temperature conditions of 24 ± 3°C. ToBRFV was mechanically inoculated onto the third true leaf. In parallel, leaf extractions from tomato plants infected with a mixture of ToBRFV and the mild PepMV-IL that showed synergism on tomato plants under mixed infection conditions [41, 42], were inoculated on test plants. PepMV-IL single inoculations were carried out as controls for quantification of the increase in PepMV-IL titer in mixed infection. At ~30 dpi, bioassays of infected *S*. *elaeagnifolium*, *S*. *rostratum* and *S*. *nigrum* plants were carried out by inoculation of *Tm-2*^*2*^ gene-bearing tomato plants. In the bioassay, fresh *S*. *rostratum* plants were also inoculated using sap of ToBRFV-inoculated *S*. *rostratum* and *S*. *lycopersicum* plants. For bioassays using *N*. *glutinosa*, equal ratios of inoculation buffer per leaf weight (6 ml/g) of *S*. *elaeagnifolium* and *S*. *rostratum* served for inoculations. Systemic ToBRFV infections showing mild mottling were tested using ELISA [19].

### Serological tests for viral infections

ELISA and western blot analyses were carried out as previously described [41]. ELISA results were considered positive when the optical density (O.D.) values were at least 2.5 times the negative (healthy) control. The negative control O.D. range was 0.005–0.015. For western blot analyses, the leaf samples were compared at constant ratios of urea-SDS-β-mercaptoethanol lysis buffer and leaf weight. Accordingly, the increase in PepMV-IL in ToBRFV and PepMV-IL mixed infected plants was a quantitative comparison with PepMV-IL singly infected plants. The specific antisera prepared against purified virions of ToBRFV from *Tm-2*^*2*^ allele-bearing tomato plants and PepMV-IL virions isolated on *D*. *stramonium* plants, as previously described [19, 42], were used in the assays. Ponceau-S staining was conducted before or following the detection of the specific coat proteins, the latter identifying the viral coat proteins (CP) and RuBisCO on the membrane.

### Viral RNA extraction and reverse transcription (RT)-PCR

Viral RNA extraction was performed on plant samples using general extraction buffer (Bioreba, Reinach, Switzerland) and the Accuprep Viral Extraction kit (Bioneer, Daejeon, Korea). cDNA was obtained using the qPCRBIO cDNA synthesis kit (PCR BIOSYSTEMS Ltd, London, UK) and PCR was performed with the following primer sets: for ToBRFV an amplicon of 611 bp was obtained with F-5557F (5′-TTTAGTAGTAAAAGTGAGAAT-3′) and R-6167R (5′-TTGTAAACCGGATGCACTTTCAAATG-3′); for PepMV-IL an amplicon of 650 bp was obtained with F-5658F (5′-CCATCAGATGCACCACCAAC-3′) and R-6307R (5′-TTAGCTCCTCCCATGTGTCC-3′). ToBRFV amplicons were Sanger sequenced (HyLabs, Rehovot, Israel) and alignments for specificity of the sequence were performed using BLAST search against NCBI GenBank (https://blast.ncbi.nlm.nih.gov/Blast.cgi).

### Quantitative RT-PCR (RT-qPCR)

Leaves from ToBRFV- or ToBRFV- and PepMV-IL- mixed-infected *S*. *elaeagnifolium*, *S*. *rostratum* and *S*. *lycopersicum* plants (50–100 mg) were subjected to total RNA extraction using a TRI Reagent kit (MRC, Inc., Cincinnati, OH, USA). Sample RNA concentrations were measured by spectrophotometer NanoDrop ND1000 (Thermo Scientific, Wilmington, DE, USA). cDNA synthesis was performed on 1 μg of total RNA using qPCRBIO cDNA synthesis kit (PCR BIOSYSTEMS Ltd, London, UK), using a combination of random hexamers with anchored oligo(dT). RT-qPCR was performed using the power SYBR Green PCR Master MIX (Applied Biosystems, Thermo Fisher Scientific, Vilnius, Lithuania) and performed using the StepOnePlusTM (Applied Biosystems, Fisher Scientific Company, Ottawa, Ontario). The endogenous gene TIP41 served as a host reference gene for *S*. *elaeagnifolium* and *S*. *lycopersicum* [42], and RPL8 served as a host reference gene for *S*. *elaeagnifolium* and *S*. *rostratum* [44]. The host genes were analyzed with each batch of virus extractions. Primers for the reference genes TIP41 and RPL8, and the target gene ToBRFV-CP, were designed with Primer3 Plus software. The primer set for ToBRFV-CP was F 5′ CACAATCGCAACTCCATCGC 3′ and R 5′ ACAGGTTTCCACACTTCGCT 3′, amplicon size 159 bp; for TIP41 primers were F 5′ ATGGAGTTTTTGAGTCTTCTGC 3′ and R 5′ GCTGCGTTTCTGGCTTAGG 3′, amplicon size 235 bp; for RPL8 primers were F 5’ CAAATCCCACACCCACCACC 3’ and R 5’ GCAACACATTACCAACCATAAGACTAGC 3’, amplicon size 260 bp. Amplification of the viruses was performed in duplicates with the specific primers. Each sample was analyzed for the presence of the TIP41 endogenous gene (*S*. *elaeagnifolium* and *S*. *lycopersicum*) or RPL8 (*S*. *elaeagnifolium* and *S*. *rostratum*). Each reaction contained 100 ng cDNA (cDNA reverse transcribed from 100 ng RNA) in a 15 μL reaction mixture containing 4 μL of diluted cDNA, 3 pmols of each primer and 7.5 μL Absolute QPCR Sybr Green Mix (Thermo Fisher Scientific, Vilnius, Lithuania). Reaction conditions were: 10 min at 95°C (hot start) followed by 40 cycles of 3 sec at 94°C, 15 sec at 60°C, and 20 sec at 72°C. The amplification efficiency of each of the samples equaled 1%. ΔCt was obtained by subtracting Ct of the endogenous gene from Ct of ToBRFV. ΔΔCt was calculated by subtracting mean ΔCt of the virus in the healthy control samples from each ΔCt of the respective infected samples. ΔΔCt of each infected sample served for calculation of 2^-ΔΔCt for estimation of relative gene expression in the infected samples, relative to the respective healthy control samples. The mean 2^-ΔΔCt ± the standard deviation of the mean (s.d.) data for the various tested samples were analyzed and graphed.

## Results

### Susceptibility of weed species to ToBRFV and PepMV-IL infections

We tested the scope of weed species potentially susceptible to ToBRFV infection, and examined two endemic weeds and nine major invasive weeds of the Israeli flora in mechanical inoculation tests (Table 1).

**Table 1:** Susceptibility of invasive and native weed species to ToBRFV and PepMV-IL infections.

Species	Family	Invasive or native	*ELISA		**RT-PCR	
			ToBRFV	PepMV-IL	ToBRFV	PepMV-IL
***Amaranthus blitoides***	*Amaranthaceae*	invasive	-	-	-	-
***Amaranthus retroflexus***	*Amaranthaceae*	invasive	-	-	-	-
***Conyza bonariensis***	*Asteraceae*	invasive	-	-	-	-
***Conyza canadensis***	*Asteraceae*	invasive	-	-	-	-
***Digitaria sanguinalis***	*Poaceae*	native	-	-	-	-
***Setaria adhaerens***	*Poaceae*	native	-	-	-	-
***Solanum nigrum***	*Solanaceae*	invasive	+	+	+	+
***Solanum rostratum***	*Solanaceae*	invasive	+	+	+	+
***Solanum elaeagnifolium***	*Solanaceae*	invasive	+	-	+	-
***Sorghum halepense***	*Poaceae*	invasive	-	-	-	-
***Xanthium strumarium***	*Asteraceae*	invasive	-	-	-	-

*Positive results in ELISA were more than 2.5 fold the negative control. **RT-PCR amplicons were confirmed by Sanger sequencing.

Two new invasive *Solanaceae* species were identified as hosts of ToBRFV. *S*. *elaeagnifolium* and *S*. *rostratum*, mechanically inoculated with ToBRFV from infected *Tm-2*^*2*^ allele-bearing tomato plants, were found infected by ToBRFV by ELISA and RT-PCR tests (Table 1).

Following the first cycle of mechanical inoculations, the experiments with *S*. *elaeagnifolium* and *S*. *rostratum* were repeated with ToBRFV, using larger sample numbers, 34 and 26 plants, respectively. In all, 35% of *S*. *elaeagnifolium* plants and 88% of *S*. *rostratum* plants were ToBRFV-positive by ELISA test. ELISA O.D. value ranges were 3–21 and 26–145 times the negative controls in *S*. *elaeagnifolium* and *S*. *rostratum* plants, respectively.

We therefore investigated whether any of the newly tested weed species were hosts of the mild PepMV-IL, which could potentially initiate synergism with the abundant ToBRFV. We found that only *S*. *rostratum*, mechanically inoculated with PepMV-IL tested positive for the virus using ELISA and RT-PCR tests (Table 1). Excluding *S*. *nigrum*, the other weeds were not hosts of PepMV-IL alone.

### *S*. *elaeagnifolium* as a host of ToBRFV

**Fig 1:**
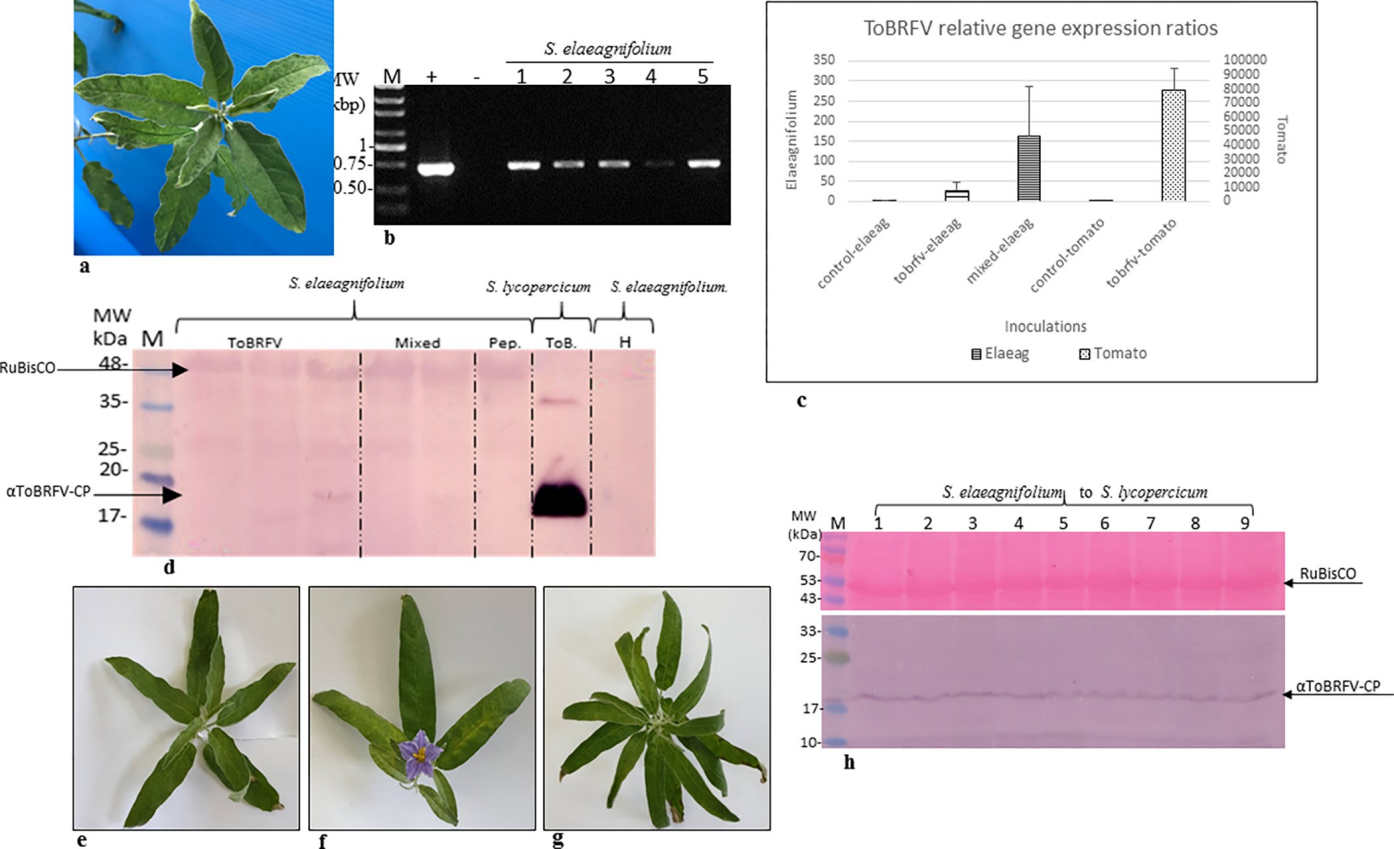
*S*. *elaeagnifolium* is a host of ToBRFV, with a low virus titer. (a) Asymptomatic ToBRFV-infected *S*. *elaeagnifolium* plants found adjacent to a commercial tomato greenhouse. (b) RT-PCR of ToBRFV-inoculated *S*. *elaeagnifolium* plants, confirmed by Sanger sequencing. (c) A graphical depiction of ToBRFV relative gene expression ratios 2^-ΔΔCt in comparison to the corresponding healthy controls (using the TIP41 endogenous gene). *S*. *elaeagnifolium* plants were inoculated with ToBRFV, or a mixture of ToBRFV and PepMV-IL from tomato plants bearing the *Tm-2*^*2*^ resistance gene. (d) Western blot analyses showing a low titer of ToBRFV-CP in *S*. *elaeagnifolium* plants compared to tomato. Plants were inoculated with ToBRFV or a mixture of ToBRFV and PepMV-IL from tomato plants. (ToBRFV n = 5, PepMV n = 2, mixed inoculation n = 5). A Ponceau-S stained gel is depicted showing both the CP and RuBisCO. (e) Depiction of a healthy control *S*. *elaeagnifolium* plant. (f) An asymptomatic *S*. *elaeagnifolium* plant 30 dpi with ToBRFV, or (g) a mixture of ToBRFV and PepMV-IL. (h) A bioassay for ToBRFV-inoculated *S*. *elaeagnifolium* plants using tomato plants. M, molecular size marker; (+) a control from ToBRFV-infected tomato plants; (-) healthy control; numbers represent different plants; arrows indicate the ToBRFV-CP and the Ponceau-S stained RuBisCO; H, a healthy control.

**Fig 2:**
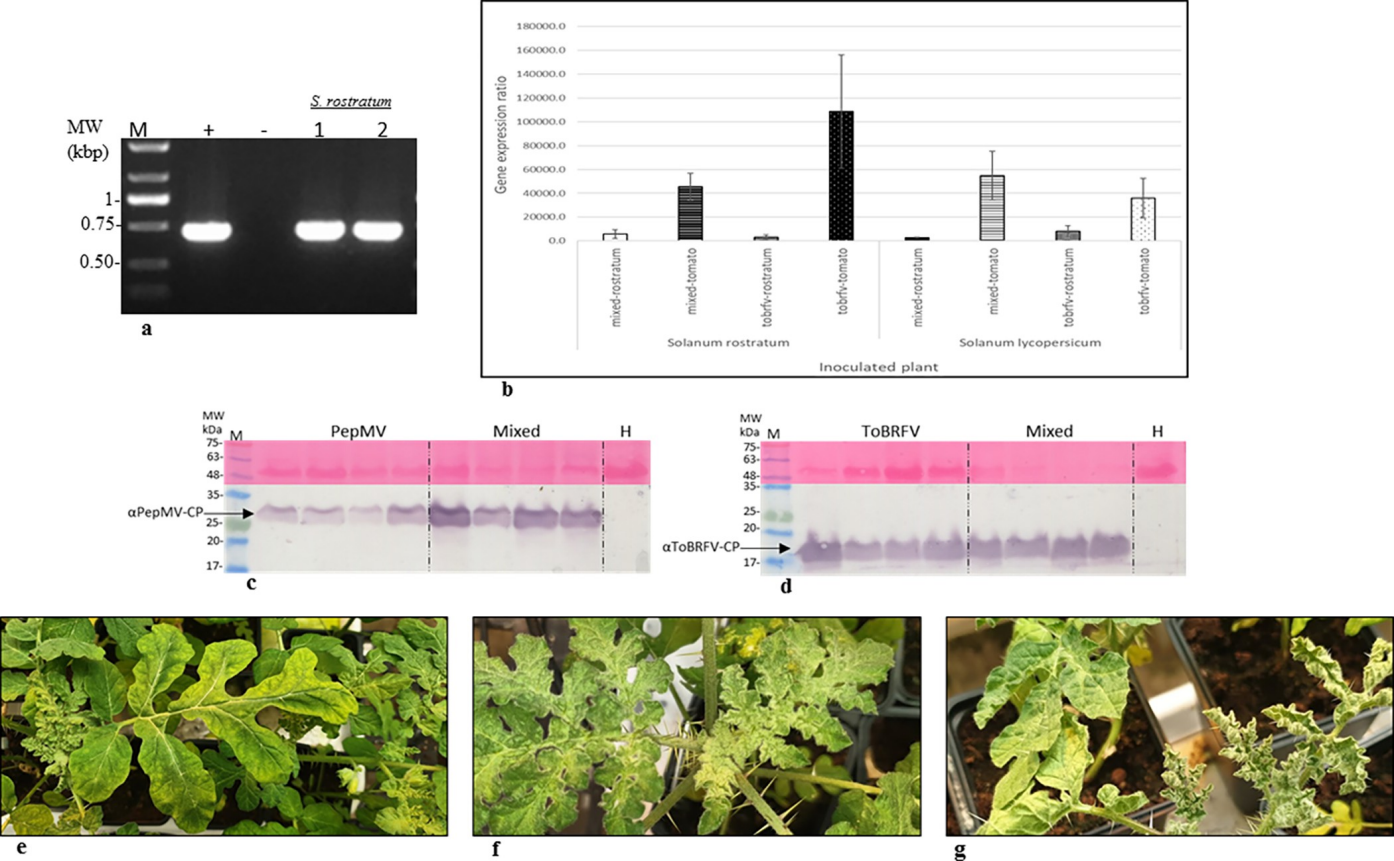
*S*. *rostratum* is a host of ToBRFV and PepMV-IL in single and mixed infections. (a) RT-PCR of ToBRFV-inoculated *S*. *rostratum* plants, confirmed by Sanger sequencing. (b) A graphical depiction of ToBRFV relative gene expression ratios 2^-ΔΔCt in comparison to the corresponding healthy controls (with the RPL8 endogenous gene). Four different inoculum sources were applied to each test plant: *S*. *rostratum* and *S*. *lycopersicum*. (c) Western blot analyses showing CP levels of PepMV-IL and (d) ToBRFV in singly and mixed-inoculated *S*. *rostratum* plants. (e) Symptomatic *S*. *rostratum* plants inoculated with PepMV-IL alone, and (f) ToBRFV alone or (g) with a mixture of ToBRFV and PepMV-IL showing leaf roll and serrated leaves. M, a molecular size marker; (+) positive control from ToBRFV-infected tomato plants; (-) healthy control; numbers represent different plants; arrows indicate the ToBRFV-CP, PepMV-CP and the Ponceau-S stained RuBisCO; H, a healthy control.

*S*. *elaeagnifolium*, found adjacent to *Tm-2*^*2*^ allele-bearing tomato plants in a commercial greenhouse were infected with ToBRFV, as determined by RT-PCR, but showed no disease symptoms (Fig 1A). *S*. *elaeagnifolium* plants were susceptible to ToBRFV infection, using tomato plants as an inoculum source (Fig 1B). To test the infectious potential of ToBRFV-inoculated *S*. *elaeagnifolium* plants, we first quantified the presence of the virus in the weed by RT-qPCR and western blot assays. ToBRFV was detected in the inoculated plants but the virus titer was very low, compared to tomato plants (Fig 1C and 1D). No phenotype was observed on ToBRFV-infected *S*. *elaeagnifolium* (Fig 2E–2G). In a bioassay performed on *N*. *glutinosa* plants with infected *S*. *elaeagnifolium*, inoculated with ToBRFV alone or with a mixture of ToBRFV and PepMV-IL, two of three test plants and one out of three test plants, respectively, were positive for ToBRFV infection. ELISA O.D. value range was 10–22 times the negative control. The infectious potential of ToBRFV-inoculated *S*. *elaeagnifolium* plants was also tested by inoculating tomato plants, which became infected by ToBRFV (Fig 1H). These results indeed confirmed that although low in ToBRFV titer, infected *S*. *elaeagnifolium* plants could serve as a primary infection source. *S*. *elaeagnifolium* plants were not hosts of PepMV-IL, tested using both a single virus inoculum source and a mixed inoculum source (from ToBRFV- and PepMV-IL-mixed infected tomato plants), in which a synergism between the co-infecting viruses augmented PepMV-IL titer [42] (Fig 1D).

### *S*. *rostratum* response to ToBRFV and PepMV-IL inoculations

*S*. *rostratum* plants inoculated with sap from ToBRFV-infected tomato plants showed susceptibility to ToBRFV infection as tested by RT-PCR, confirmed by Sanger sequencing (Fig 2A). Unlike *S*. *elaeagnifolium*, *S*. *rostratum* plants could be inoculated with ToBRFV alone, or a mixture of ToBRFV and PepMV-IL containing a high ToBRFV titer when the inoculum source was *S*. *lycopersicum* plants (Fig 2B, left panel). In a biological assay using *N*. *glutinosa* plants, three of three plants were ToBRFV-positive; ELISA O.D. value range was 16–19 times the negative controls. We also tested the infectious potential of ToBRFV-infected *S*. *rostratum* plants by either inoculating healthy *S*. *rostratum* or *S*. *lycopersicum* plants (Fig 2B, left and right panels). Both test plants were infected. Nevertheless, the tomato inoculum source constantly induced a higher ToBRFV titer in the inoculated plants than did the *S*. *rostratum* source (Fig 2B). Similar to *S*. *lycopersicum* plants, *S*. *rostratum* plants were infected by PepMV-IL upon single inoculation or mixed inoculation with ToBRFV. Moreover, in *S*. *rostratum* a synergism between the viruses occurred with an increased PepMV-IL titer on mixed infection (Fig 2C and 2D). The synergism was also observed in symptom development showing yellowing and mottling of serrated leaves and leaf roll (Fig 2W–2G).

To confirm the preservation and infectious potential of ToBRFV in *S*. *rostratum*, which reproduces only via seeds, we planted four ToBRFV-infected plants in 100 L pots for flowering and seed development under natural environmental conditions. One of the five mature plants grown from the germinated seeds was infected with ToBRFV in ELISA test, showing O.D. values of 4.7 times the negative reference with no visual symptoms.

### *S*. *nigrum* shows synergism upon mixed infections with ToBRFV and PepMV-IL

**Fig 3:**
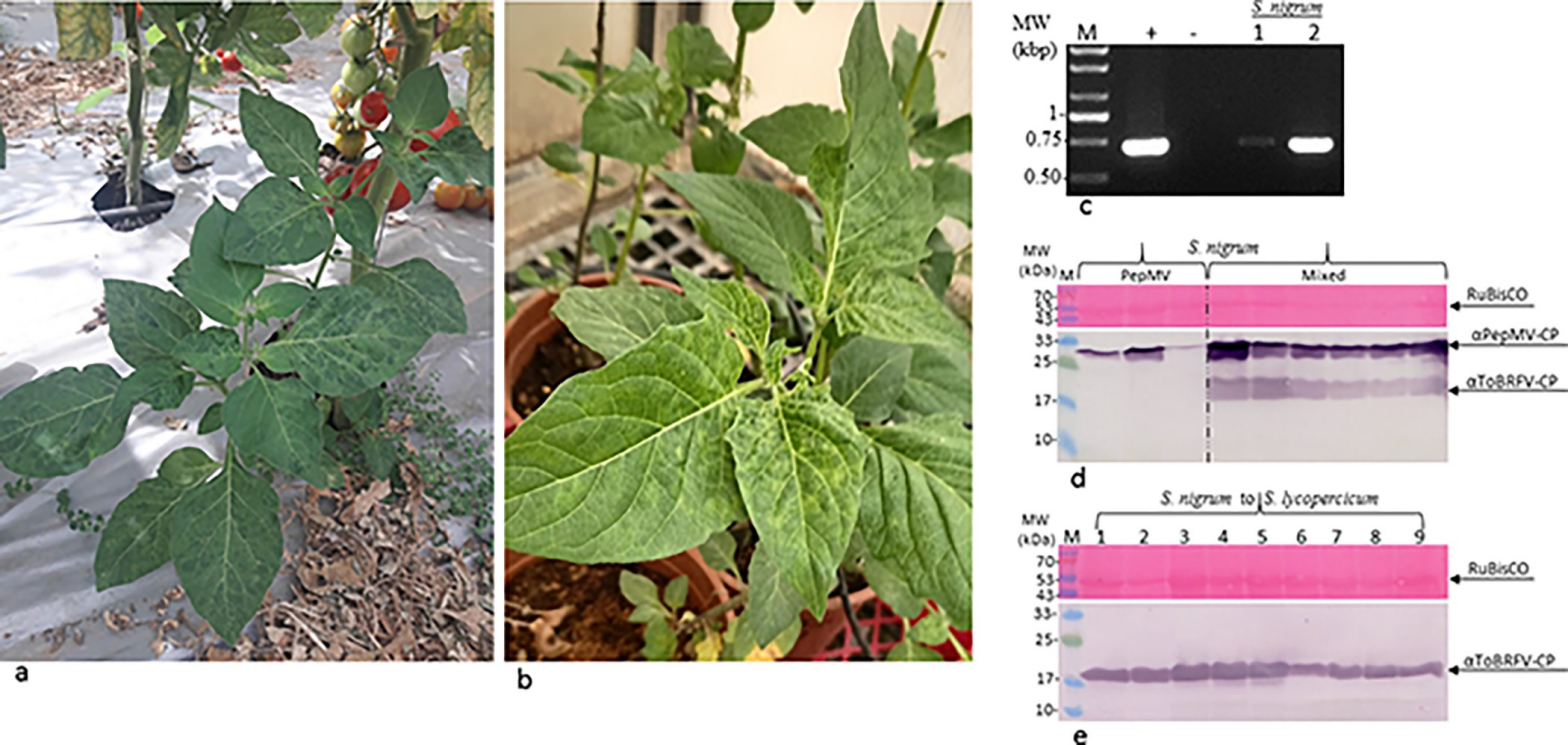
*S*. *nigrum* shows synergism upon mixed infections with ToBRFV and the mild strain PepMV-IL. (a) ToBRFV-infected naturally occurring *S*. *nigrum* showing mottled mosaic leaves grew adjacent to tomato plants in a commercial tomato greenhouse. (b) A ToBRFV-inoculated *S*. *nigrum* plant showing mild mottled leaves. (c) RT-PCR of ToBRFV-infected *S*. *nigrum*, confirmed by Sanger sequencing. (d) A western blot analysis showing a viral synergism in *S*. *nigrum* plants upon mixed infection with ToBRFV and PepMV-IL. (e) A bioassay of ToBRFV-inoculated *S*. *nigrum* plants using tomato plants bearing the *Tm-2*^*2*^ resistance gene. M, molecular size marker; (+), a positive control for ToBRFV from tomatoes; (-) a healthy control; numbers represent different plants inoculated with ToBRFV.

We found *S*. *nigrum* plants growing adjacent to tomato crops in commercial greenhouses, these weeds showed mottled mosaic leaves and tested positive for ToBRFV by RT-PCR (Fig 3). We confirmed susceptibility of *S*. *nigrum* plants to ToBRFV infection, using an inoculum from ToBRFV-infected tomato plants and analysis by both ELISA and RT-PCR (Table 1, Fig 3). ELISA O.D. value range was 20–38 times the healthy controls. In addition, we tested the plants for susceptibility to infection by the mild PepMV-IL. PepMV-IL infected *S*. *nigrum* plants and a viral synergism occurred on co-infection with ToBRFV and the mild PepMV-IL, as shown in a western blot (Fig 3D). In a bioassay, ToBRFV-inoculated *S*. *nigrum* plants infected tomato plants bearing the *Tm-2*^*2*^ resistance gene (Fig 3E).

## Discussion

Weeds growing adjacent to crops could serve as reservoirs of disease-causing agents counteracting crop rotations and quarantines implemented to contain diseases. We identified two new invasive weeds *S*. *elaeagnifolium* and *S*. *rostratum* as hosts of ToBRFV and confirmed the potential of these plants and the previously identified ToBRFV host plant *S*. *nigrum* [19] as ToBRFV reservoirs. Due to the threat of synergistic effects occurring between ToBRFV and a mild PepMV-IL strain, we tested the weeds for a possible viral synergism, and found that *S*. *nigrum* and *S*. *rostratum* both showed a synergism when co-infected with these viruses (Figs 2 and 3). PepMV-IL was not detected in *S*. *elaeagnifolium* plants (Fig 1).

*S*. *elaeagnifolium* is a deep-rooted perennial weed species native to the Western plains of the United States and Mexico [45]. According to A. Dafni, the initial introduction of this species into Israel occurred in 1956 through Egypt [46]. Today, this weed has spread strongly across Israel and inhabits extensive areas and multiple habitats. Due to its abundance across the country, we may assume that since the first introduction in 1950s multiple introductions have occurred. Propagation of this weed occurs *via* seeds, creeping rhizomes or root fragments [47]. Root fragments retain high sprouting ability and could extend up to 2 m from the parent plant [48]. In addition, dried plants could break off and spread their attached berries with the wind, similar to tumbleweed seed dispersal [46]. In Israel, *S*. *elaeagnifolium* infests agricultural and non-agricultural habitats including field crops, roadsides and waste grounds. Several studies have indicated *S*. *elaeagnifolium* potential as a host of crop plant pests. *S*. *elaeagnifolium* was identified as a natural host of tomato yellow leaf curl virus and tomato yellow leaf curl Sardinia virus [49]. In addition, *S*. *elaeagnifolium* plants collected from pepper (*Capsicum annum*) fields were identified as hosts of pepper mottle virus [50].

We have found that although *S*. *elaeagnifolium* had a low ToBRFV titer, the plant had an infectious potential on *N*. *glutinosa* plants, and tomato plants bearing the *Tm-2*^*2*^ resistance gene. The low ToBRFV titer could be related to the genetic background of *S*. *elaeagnifolium*, which has a close genetic proximity to *S*. *melongena* [51], which was not susceptible to ToBRFV infection [19, 38]. Although PepMV-IL infection was undetectable, *S*. *elaeagnifolium* plants showed a higher titer of ToBRFV when the inoculum source was mixed-infected tomato plants compared to singly infected tomato plants (Fig 1). This could be the result of a high ToBRFV titer in the mixed infected inoculum as has been previously observed in tomato plants grown at 25°C [42] which could be indicative of an unknown role of PepMV in stabilizing ToBRFV titer. The lack of symptom development of ToBRFV-infected *S*. *elaeagnifolium* plants could indicate that host defense responses could be more effective under conditions of low ToBRFV systemic infections, determined by host-restricted susceptibility to ToBRFV replication or movement.

Unlike *S*. *elaeagnifolium* plants, *S*. *rostratum* susceptibility to ToBRFV and PepMV-IL infections was similar to that of tomato. These responses could be an additional indication of genetic similarities between the two *Solanum* species with a possible common ancestor [52, 53].

*S*. *rostratum*, a noxious weed native of the Mexican highlands [54], has invaded several different regions across the world including Canada, China, Russia, Australia, and Europe [55]. This species reproduces only *via* seeds, as dried berries open up and spread their seeds. However, similar to *S*. *elaeagnifolium*, plants may also break and move across the land as tumbleweeds, improving seed distribution [54]. In Israel, *S*. *rostratum* was first found in the Jezreel valley in 1953, and since then has been located in the Jordan valley, the Hula valley and along the Mediterranean Sea coastline [56]. *S*. *rostratum* can be found mainly within field and at field crop margins, such as watermelon (*Citrullus lanatus*) and tomatoes. Although the infectious potential of ToBRFV in *S*. *rostratum* was lower than that of ToBRFV in *S*. *lycopersicum* (Fig 2B), an ongoing evolutionary process is possible when further viral host jumps may accelerate the spread and the damage of the virus in the future via *S*. *rostratum*. The severe disease symptom manifestations associated with ToBRFV and PepMV-IL mixed infections of *S*. *rostratum* could serve as a warning sign designating a disease area detrimental for re-growing tomato crops.

## Conclusions

Several studies have indicated potential of the *S*. *elaeagnifolium* as a host of crop pests. However, to the best of our knowledge, this is the first report on *S*. *rostratum* as a host of tobamoviruses and potexviruses, and of the synergism between ToBRFV and PepMV-IL in the weeds *S*. *rostratum* and *S*. *nigrum*. The distribution and abundance of these weed species within and in close proximity to agricultural fields in general, and to tomato fields in particular, increase the risks of virus transmission between species. The unique response of *S*. *elaeagnifolium* to inoculations with ToBRFV could indicate that the unique plant defense response determines the low virus titer as well as phenotype preservation and it is worth further studying as a tool for the development of virus-resistant varieties.

## Supporting information

S1 Raw images (TIF)

S2 Raw images (TIF)

S3 Raw images (TIF)
