# Supplementary figures and images for: Solanum elaeagnifolium and S. rostratum as potential hosts of the tomato brown rugose fruit virus
Source: PLoS One. 2023 Mar 1;18(3):e0282441. doi: 10.1371/journal.pone.0282441 (PMC9977001; doi:10.1371/journal.pone.0282441)

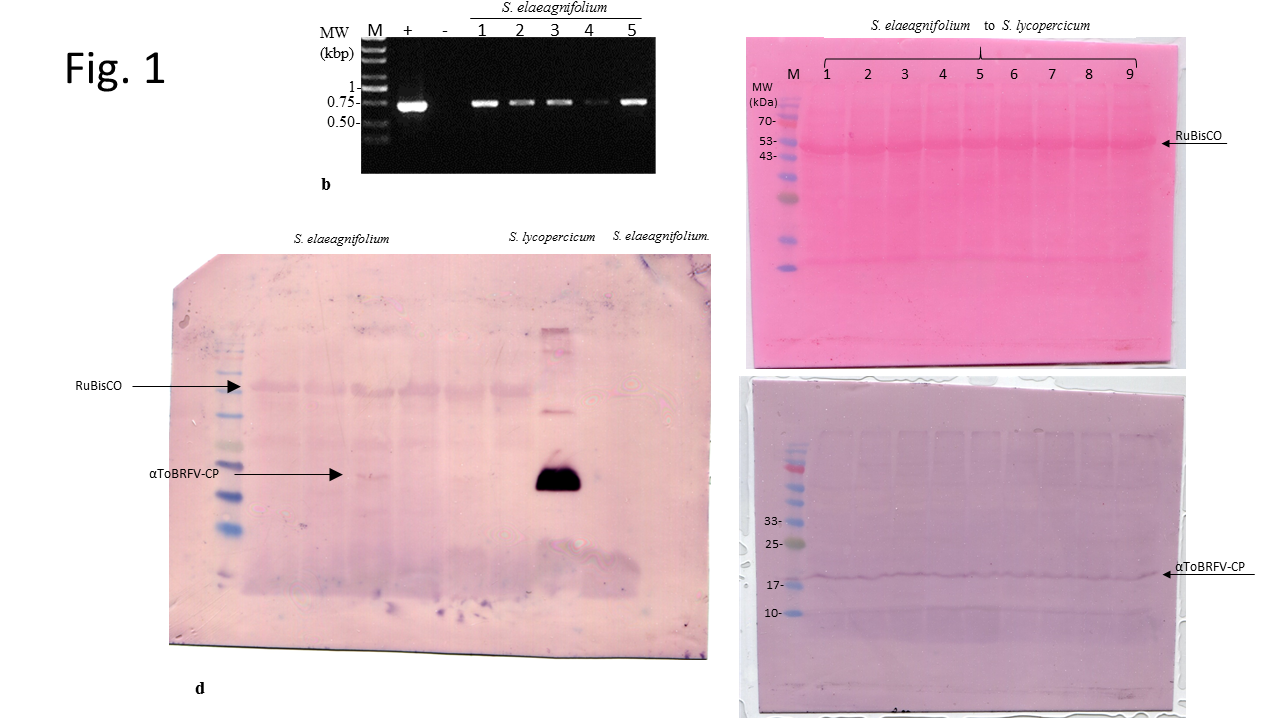

Supplement: S1 Raw images — (TIF) [file pone.0282441.s001.tif]

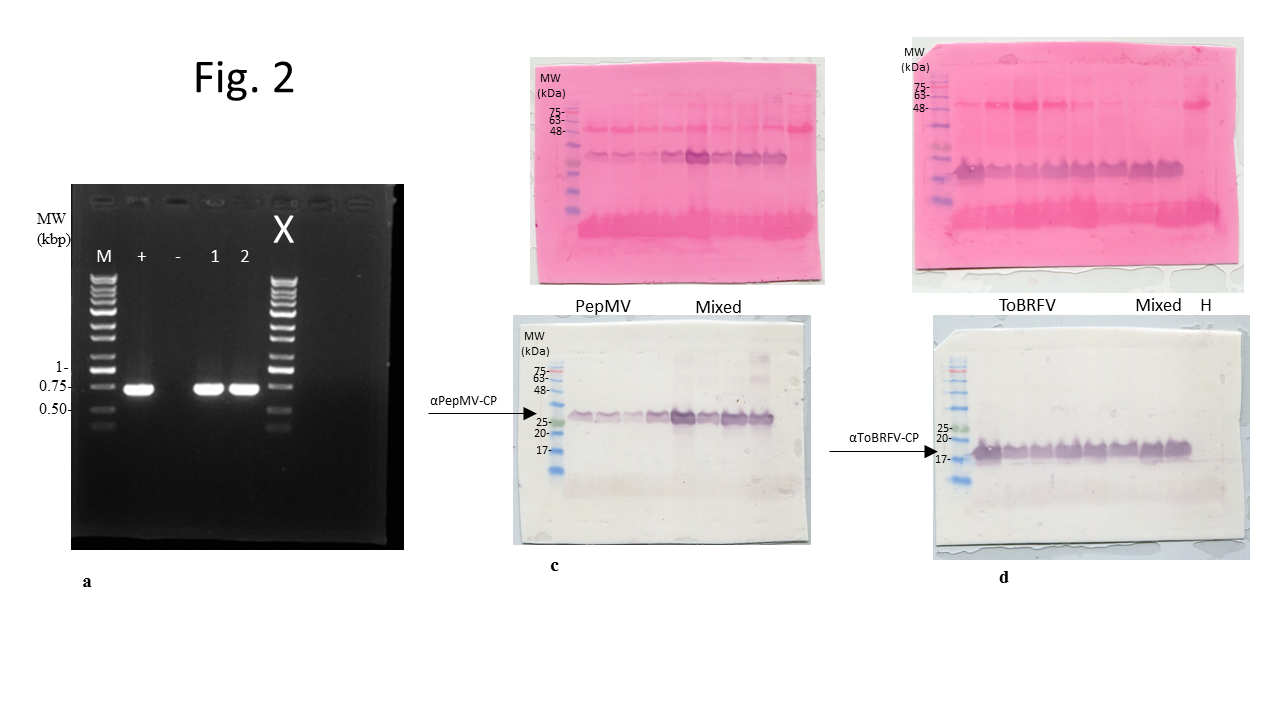

Supplement: S2 Raw images — (TIF) [file pone.0282441.s002.tif]

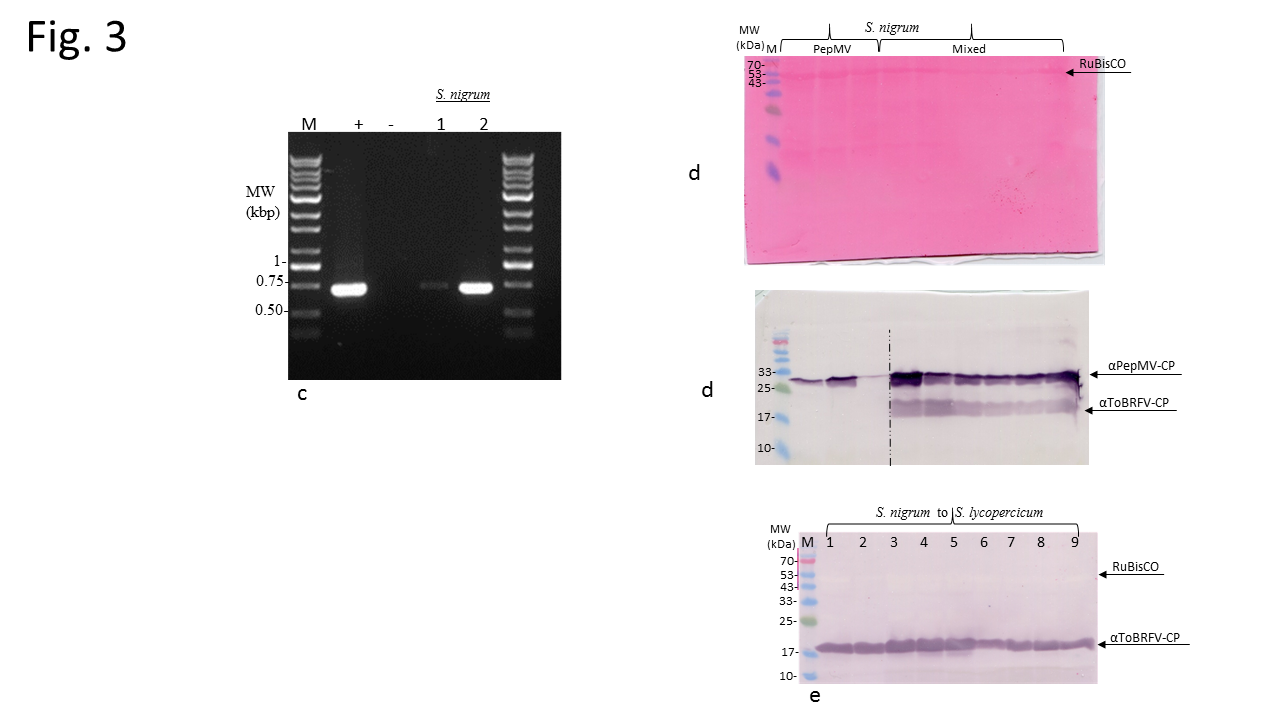

Supplement: S3 Raw images — (TIF) [file pone.0282441.s003.tif]
